# Supplementary material for: The lymphocyte-specific protein tyrosine kinase-specific inhibitor A-770041 attenuates lung fibrosis via the suppression of TGF-β production in regulatory T-cells
Source: PLoS One. 2022 Oct 27;17(10):e0275987. doi: 10.1371/journal.pone.0275987 (PMC9612470; doi:10.1371/journal.pone.0275987)
Supplement: S1 File — (DOCX) [file pone.0275987.s001.docx]

**The lymphocyte-specific protein tyrosine kinase-specific inhibitor A-770041 attenuates lung fibrosis via the suppression of TGF-β production in regulatory T-cells**

Kozo Kagawa^1^, Kazuya Koyama^1^, Seidai Sato^1^, Takeshi Imakura^1^, Kojin Murakami^1^, Yuya Yamashita^1^, Nobuhito Naito^1^, Hirohisa Ogawa^2^, Hiroshi Kawano^1^, Yasuhiko Nishioka^1^.

^1^Department of Respiratory Medicine and Rheumatology, Graduate School of Biomedical Sciences, Tokushima University, Tokushima, Japan

^2^Department of Pathology and Laboratory Medicine, Graduate School of Biomedical Sciences, Tokushima University, Tokushima, Japan

**SUPPLEMENTAL DATA**

**Material and Methods**

***Mice and agents***

Eight-week-old C57BL/6 male mice were purchased from Charles River Japan Inc (Tokyo, Japan). The mice were maintained in the animal facility of the Tokushima University under specific-pathogen-free conditions according to the guidelines of Tokushima University. All experimental protocols were approved by the animal research committee of the University of Tokushima, Japan. A-770041 and Nintedanib were kindly provided by Boehringer Ingelheim (Ingelheim, Germany). Antibodies for Lck and phospho-Lck were purchased from Cell Signaling Technology (Danvers, MA, USA). Anti-actin β was purchased from Santa Cruz Biotechnology (Santa. Cruz, CA, USA).

***CD4^+^ T-cell isolation***

CD4^+^ T-cells were obtained from murine lung or spleen. Spleens were minced to obtain splenocytes. After mashing through 100-μm cell strainers, CD4^+^ T-cells were isolated by using auto-MACS (Miltenyi Biotec K.K., Bergisch Gladbach, Germany) with CD4^+^ T Cell Isolation Kits (Miltenyi Biotec K.K.).

***Treg cell isolation***

Treg cells were obtained from murine spleen. Spleens were minced to obtain splenocytes. After mashing through 100-μm cell strainers, Treg cells were isolated by using auto-MACS (Miltenyi Biotec K.K., Bergisch Gladbach, Germany) with CD4^+^ CD25^+^ Regulatory T Cell Isolation Kits (Miltenyi Biotec K.K.).

***Lung single cell suspension***

Murine lungs were minced and digested with DNase I (Roche, Branford, CT, USA) and Collagenase I (Roche) for 1 h at 37°C to obtain single cell suspensions.

***Analyses of the phosphorylation of Lck***

CD4^+^ T-cells were incubated with beads coated with CD3/CD28 antibody (T cell activation expansion kit, Miltenyi Biotec K.K) and with RPMI with various concentration of nintedanib (0, 3,10, 30, 100, 300 nM) or A-770041 (0, 30, 100, 300, 1000 nM) for 5 minutes. After stimulation with CD3/CD28 antibody, CD4^+^ T-cells were collected and lysed immediately, and the phosphorylation of Lck was determined using the Simple WesternTM System (ProteinSimple, Santa Clara, CA, USA) according to a previous report [E1].

***Simple WesternTM System***

Whole-cell extracts were prepared with M-PER reagents (Thermo Fisher Scientific) containing phosphatase and protease inhibitor cocktails (Roche). Protein concentrations were measured using the Bradford method. The same amounts of total cell extracts proteins were used for the Simple Western^TM^ System (ProteinSimple, Santa Clara, CA, USA). We used the Simple Western^TM^ System as described in a previous report [E1] and according to the manufacturer’s instructions, and we analyzed the protein amounts based on the signal intensity.

***Bleomycin-induced lung fibrosis in mice***

Mice received a single transbronchial instillation of 3.0 mg/kg BLM on day 0 as previously described [E2]. A-770041 (5 mg/kg), nintedanib (60 mg/kg) or distilled water was administered daily by gavage. The dosage of A-770041 was determined based on previous study [E3].

***The hydroxyproline assay***

Twenty-one days after BLM instillation, the lungs were harvested and homogenized in distilled water, and the hydroxyproline contents were measured using a Bio-vision hydroxyproline assay kit (BioVision, Mount View, CA, USA).

***Histopathology***

BLM-treated lungs were harvested, fixed in 10% formalin, and embedded in paraffin. Three-micrometer-thick sections were stained with hematoxylin and eosin (H&E) stain or azan Mallory. In the quantitative analysis, a numeric fibrotic scale was used (Ashcroft score) [E4]. The mean score was considered to be the fibrotic score.

***Bronchoalveolar lavage***

Mice were anesthetized, and a soft cannula was inserted into the trachea. Bronchoalveolar lavage (BAL) was performed from both lungs with saline (1 ml) at various time points. The total cell count of the BAL fluid was determined using Turk staining solution. The BAL fluid was centrifuged, and the cell pellets were re-suspended into saline and then cytospined onto glass slides. These cells were stained with Diff-Quick staining solution (Baxter), and 200 cells were counted for cell classification [E5]. Supernatants were collected and their cytokine concentrations were analyzed as described below.

***Flow cytometric analysis of CD4^+^ T-cells and CD8^+^ T-cells***

After the cells from BALF were meshed through 100 μM cell strainers, the cells were stained with conjugated antibodies (Abs). Followed by incubating with anti-CD16/CD32 to block Fc receptors before staining, the cells were stained with Abs to CD45 FITC, CD3e BUV395, CD4 BUV737 and CD8 PE. Anti FITC-conjugated CD45 Ab and anti PE-conjugated CD8 Ab were purchased from BioLegend (San Diego, CA, USA), and anti BUV395-conjugated CD3e Ab and anti BUV737-conjugated CD8 Ab were purchased from BD Biosciences (San Jose, CA, USA). Stained cells were analyzed by BD LSRFortessa (BD Biosciences, San Diego, CA, USA). CD4^+^ T cells were identified as CD45^+^ CD3^+^ CD4^+^ cells and CD8^+^ T-cells were identified as CD45^+^ CD3^+^ CD8^+^ cells.

***Flow cytometric analysis of IL17^+^ CD4^+^ T-cells and TGF-β1^+^ CD4^+^ T-cells***

After the cells from lung single cell suspensions were meshed through 100 μM cell strainers, the cells were stained with conjugated antibodies (Abs). Followed by incubating with anti-CD16/CD32 to block Fc receptors, the cells were stained with Abs to CD3e BUV395 and CD4 BUV737. The cells were permeabilized with Fixation/Permeabilization Kit (BD Biosciences, San Diego, CA, USA) according to the manufacturer’s instructions, and stained with Abs to IL-17 PE-Cy7 and TGF-β1 PE. Anti PE-Cy7-conjugated IL-17 Ab and anti PE-conjugated TGF-β1 Ab were purchased from BioLegend (San Diego, CA, USA), and anti BUV395-conjugated CD3e Ab and anti BUV737-conjugated CD4 Ab were purchased from BD Biosciences (San Jose, CA, USA). Stained cells were analyzed by BD LSRFortessa (BD Biosciences, San Diego, CA, USA). IL17^+^ CD4^+^ T-cells were identified as CD3^+^ CD4^+^ IL17^+^cells and TGF-β1^+^ CD4^+^ T-cells were identified as CD3^+^ CD4^+^ TGF-β1^+^cells.

***Flow cytometric analysis of Treg cells and TGF-β1^+^ Treg cells***

After the cells from lung single cell suspensions were meshed through 100 μM cell strainers, the cells were stained with conjugated antibodies (Abs). Followed by incubating with anti-CD16/CD32 to block Fc receptors, the cells were stained with Abs to CD3e APC-Cy7 and CD4 PE-Cy7. The cells were permeabilized with Fixation/Permeabilization Kit (BD Biosciences, San Diego, CA, USA) according to the manufacturer’s instructions, and stained with Abs to Foxp3 FITC and TGF-β1 PE. Anti APC-Cy7-conjugated CD3 Ab, anti PE-Cy7-conjugated CD4 Ab, anti FITC-conjugated Foxp3 Ab and anti PE-conjugated TGF-β1 Ab were purchased from BioLegend (San Diego, CA, USA). Stained cells were analyzed by BD FACSVerse (BD Biosciences, San Diego, CA, USA). Treg cells were identified as CD3^+^ CD4^+^ Foxp3^+^cells and TGF-β1^+^ Treg cells were identified as CD3^+^ CD4^+^ Foxp3^+^ TGF-β1^+^cells.

***Analyses of the expression and production of TGF-β in Treg cells.***

Treg cells were incubated with beads coated with CD3/CD28 antibody (T cell activation expansion kit, Miltenyi Biotec K.K) and with RPMI with various concetration of A-770041 (100, 500 nM) for 24 hours. Supernatants were collected and used for ELISA assay to analyze TGF-β concentration as described below. To evaluate the mRNA expression of *Tgf*b*,* cells were also lysed and used for RT-PCR as described below.

***TGF-β concentrations***

The concentrations of TGF-β of BAL fluids and supernatants of cell cultures were examined using the ELISA kit which purchased from R&D Systems (Minneapolis, MN, USA).

***Quantitative PCR***

Quantitative PCR was performed as previously described [E6]. Total RNA was extracted from Treg cells using a RNeasy Mini Kit (Qiagen, Valencia, CA), and was reverse-transcribed to cDNA using a High-Capacity cDNA Reverse Transcription Kit (Applied Biosystems, Carlsbad, CA) according to the manufacturer’s instructions. RT-PCR was performed using the CFX96 real-time PCR system (Bio-Rad, Hercules, CA) and the SYBR Premix Ex Taq (TAKARA, Kyoto, Japan). murine *GAPDH* mRNA was used as housekeeping genes, and quantification was performed using the ∆∆ Ct method. The sequences of primers were as follows: Tgfb1 forward, *5*’-CTGCTGACCCCCACTGATAC-3’, Tgfb1 reverse, 5’-GTGAGCGCTGAATCGAAAGC-3’, Gapdh forward, 5’-TCACCACCATGGAGAAGGC-3’, Gapdh reverse, 5’-GCTAAGCAGTTGGTGGTGCA-3’.

***Statistical analyses***

The significance of differences was analyzed using the one-way ANOVA, followed by Tukey’s multiple-comparison post-hoc test. *p* values of less than 0.05 were considered to be significant. Statistical analyses were performed using the GraphPad Prism software program Ver. 6.01 (GraphPad Software Inc., San Diego, CA, USA).

**References**

[E1] Chen JQ, Heldman MR, Herrmann MA, Kedei N, Woo W, Blumberg PM, et al. Absolute quantitation of endogenous proteins with precision and accuracy using a capillary Western system. Anal Biochem. 2013; 442: 97-103.

[E2] Sato S, Shinohara S, Hayashi S, Morizumi S, Abe S, Okazaki H, et al. Anti-fibrotic efficacy of nintedanib in pulmonary fibrosis via the inhibition of fibrocyte activity. Respir Res. 2017; 18: 172.

[E3] Burchat A, Borhani DW, Calderwood DJ, Hirst GC, Li B, Stachlewitz RF. Discovery of A-770041, a src-family selective orally active lck inhibitor that prevents organ allograft rejection. Bioorg Med Chem Lett. 2006; 16: 118-22.

[E4] Ashcroft T, Simpson JM, Timbrell V. Simple method of estimating severity of pulmonary fibrosis on a numerical scale. J Clin Pathol. 1988; 41: 467-70.

[E5] Aono Y, Kishi M, Yokota Y, Azuma M, Kinoshita K, Takezaki A, et al. Role of platelet-derived growth factor/platelet-derived growth factor receptor axis in the trafficking of circulating fibrocytes in pulmonary fibrosis. Am J Respir Cell Mol Biol. 2014; 51: 793-801.

[E6] Mitsuhashi A, Goto H, Saijo A, Trung VT, Aono Y, Ogino H, et al. Fibrocyte-like cells mediate acquired resistance to anti-angiogenic therapy with bevacizumab. Nat Commun. 2015; 6: 8792.
