# Supplementary figures and images for: The lymphocyte-specific protein tyrosine kinase-specific inhibitor A-770041 attenuates lung fibrosis via the suppression of TGF-β production in regulatory T-cells
Source: PLoS One. 2022 Oct 27;17(10):e0275987. doi: 10.1371/journal.pone.0275987 (PMC9612470; doi:10.1371/journal.pone.0275987)

# S1 Fig

## (a) pLck

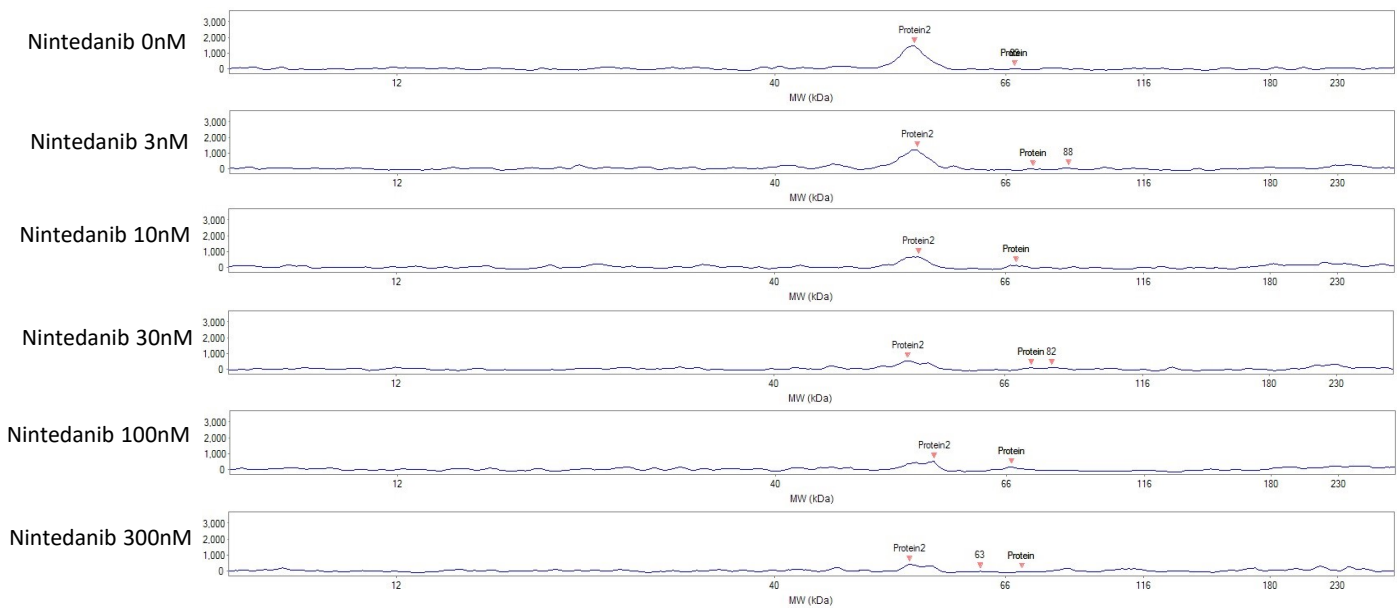

## (b) Lck

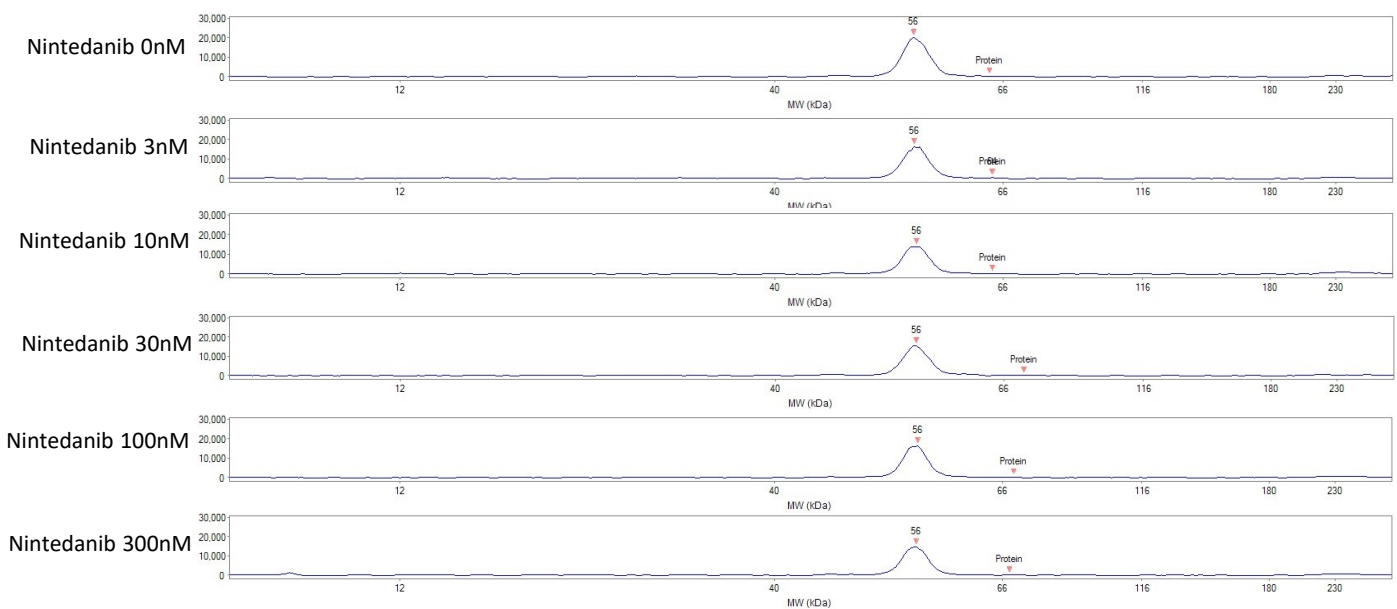

## (c) $\beta$ -actin

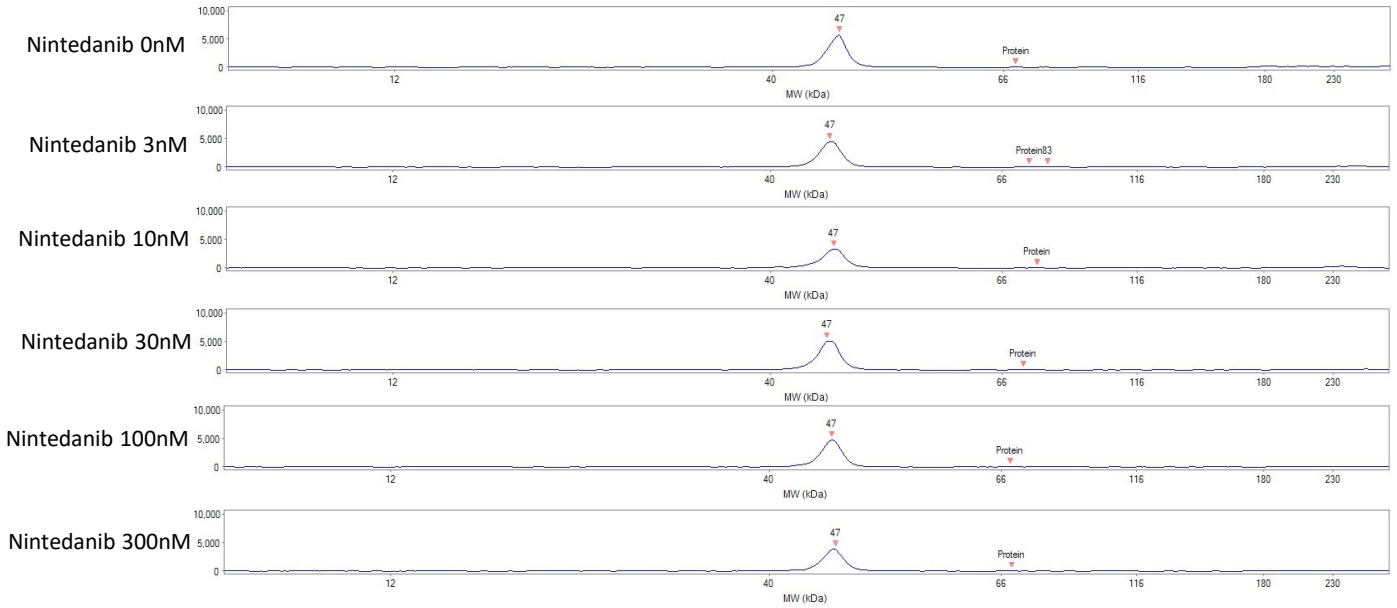

Supplement: S1 Fig — Phosphorylated Lck (a), Lck (b) and β-actin (c) were determined by a Simple WesternTM System. The vertical axis of each graph shows the fluorescence intensity of each molecular weight protein. (PDF) [file pone.0275987.s002.pdf]

S2 Fig

(a) pLck

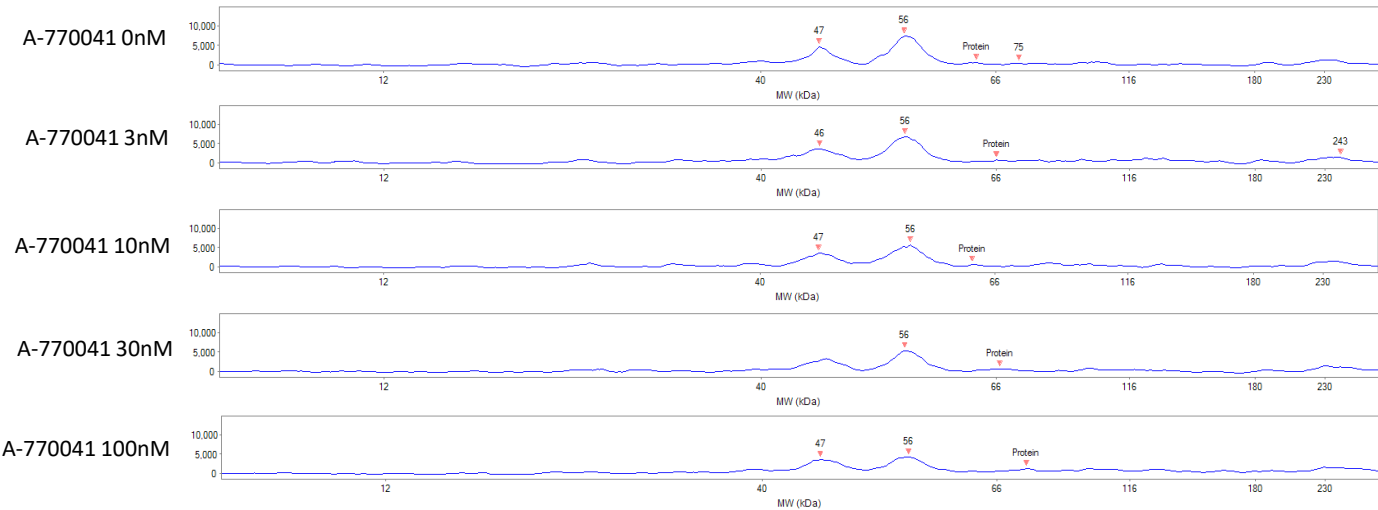

(b) Lck

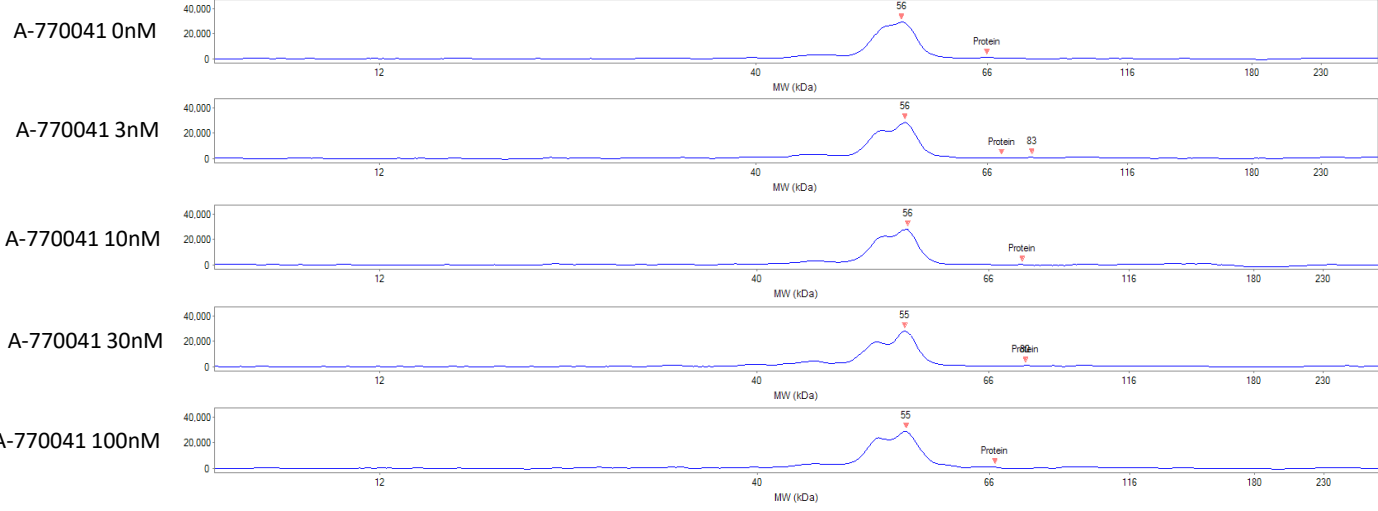

(c)  $\beta$ -actin

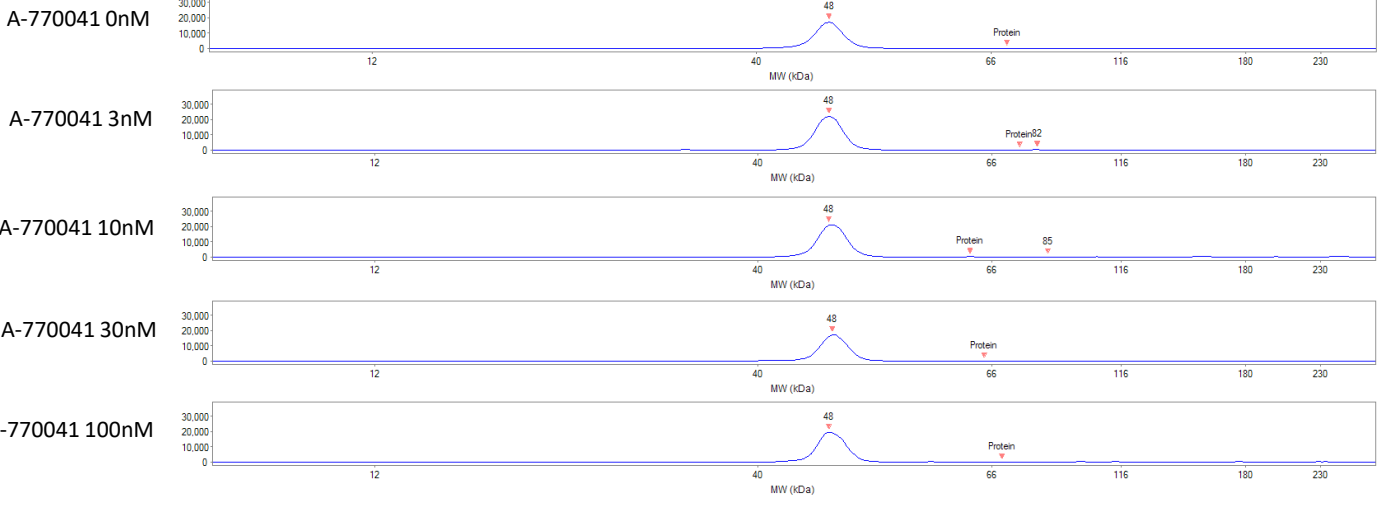

Supplement: S2 Fig — Phosphorylated Lck (a), Lck (b) and β-actin (c) were determined by a Simple WesternTM System. The vertical axis of each graph shows the fluorescence intensity of each molecular weight protein. (PDF) [file pone.0275987.s003.pdf]
